# Evaluation of color stability on resin composite specimens using different polishing kits: an *in vitro* study

**DOI:** 10.3389/fdmed.2025.1669071

**Published:** 2025-09-23

**Authors:** Hamed S. Alghamdi, Hanan Z. Alosaimi, Demah E. Kaki, Amani H. Majrashi, Ayah I. Rassas, Ghena AlHumaid, Turki Alshehri, Abdulrahman A. Balhaddad, Mohammed A. Barashi, Rashed A. Alsahafi, Hesham A. Alhazmi

**Affiliations:** ^1^ Oral and Maxillofacial Surgery Department, College of Dental Medicine, Umm Al-Qura University, Makkah, Saudi Arabia; ^2^ College of Dentistry, Umm Al-Qura University, Makkah, Saudi Arabia; ^3^ College of Dentistry, Imam Abdulrahman Bin Faisal University, Dammam, Saudi Arabia; ^4^ Department of Substitutive Dental Sciences, College of Dentistry, Imam Abdulrahman Bin Faisal University, Dammam, Saudi Arabia; ^5^ Department of Restorative Dental Sciences, College of Dentistry, Imam Abdulrahman Bin Faisal University, Dammam, Saudi Arabia; ^6^ Preventive Dentistry Department, College of Dental Medicine, Umm Al-Qura University, Makkah, Saudi Arabia; ^7^ Department of Restorative Dental Sciences, College of Dentistry, Umm Al-Qura University, Makkah, Saudi Arabia

**Keywords:** color stability, finishing, polishing, aesthetic restorations, resin composite

## Abstract

**Objectives:**

Modern dentistry has introduced color-matching restorations along with commercial finishing/polishing kits to improve color stability. This study aims to evaluate the effect of three finishing and polishing kits on the color stability of Filtek™ Z250 resin composite.

**Methods:**

This study involved 140 resin composite discs categorized into three experimental groups: Sof-Lex, Super-Snap, and OptiDisc (40 discs each), along with a control group of 20 discs. Each disc was exposed to either coffee or cola after finishing or following both finishing and polishing. Mean color change was computed for each group. A One-Way ANOVA was conducted to compare mean color changes among the finishing kits.

**Results:**

Super-Snap demonstrated the highest color stability among different finishing kits for Filtek™ Z250 resin composites in coffee staining, with a mean color change of 10.7. After finishing and polishing, its color stability improved to 10.2. For cola staining, Super-Snap had a mean color stability of 2.98, which decreased to 2.07 after polishing. However, there were no significant differences between the kits (*p* > 0.05). Furthermore, immersion in coffee resulted in significantly greater color changes compared to cola for all tested groups (*p* < 0.05).

**Conclusion:**

The three multi-step finishing and polishing kits for Filtek™ Z250 demonstrated similar color stability, with coffee showing a higher staining potential than cola. Patients with anterior composite restorations may require education about the risk of staining and the importance of dental follow-ups to maintain the aesthetic quality of their restorations.

## 1 Introduction

In modern dental practice, ensuring an accurate color match is important, as is reflected by the rising demand for aesthetically pleasing restorations (1). Composite resins are just one of the preferred materials for these kinds of restorations in both anterior and posterior teeth due to their aesthetic and mechanical properties (2). With demand in the dental field increasing continuously, composite resins have evolved to meet this demand. The success of composite resin restorations is determined by their resistance to color changes and the quality of their finishing and polishing (1). Color stability is defined as a “dental material's ability to retain its original color.” The stability of color in composite resins is influenced by various factors, including the size and number of fillers, the level of polymerization achieved, and the resins' ability to absorb water. In cases when composite restorations fail, marginal stains are a primary factor, potentially resulting in a patient's dissatisfaction and the need for replacement of the restoration (3).

Extrinsic factors contribute to negative effects on the esthetic quality of composite resin materials. Extrinsic factors, such as food particles, beverages, and increased use of colored mouth rinses (e.g., chlorhexidine) have been documented as having the most pronounced effect on colors' stability and the long-term esthetics of composite restorations (4). The final aesthetic outcome and aging of tooth-colored restorative materials rely heavily on the accuracy and quality of the finishing and polishing techniques applied. High-quality finishing and polishing enhance aesthetics and extend the longevity of composite restorations. In contrast, rough and poorly polished surfaces can lead to staining, plaque accumulation, recurrent caries, and surface porosity-related discoloration (5–8).

The purpose of finishing a restoration's shapes and contours is to replicate the tooth's natural anatomy, while polishing smooths the tooth's surface. Because finishing instruments often leave fine scratches, a variety of systems have been developed to achieve a smooth surface and reduce the risk of secondary caries. These systems include aluminum oxide-coated discs, rubber cups and points, diamond-coated burs, polishing pastes, abrasive strips, and wheels with different abrasive grits. Each system offers unique levels of smoothness (9). Among the most used finishing and polishing systems are Sof-Lex™, OptiDisc™, and Super-Snap™, due to their versatility, ease of use, and consistent clinical results (10). The Solflex™ finishing and polishing discs use color-coded grit sequences and snap onto a mandrel via a small eyelet, thereby allowing quick and alignment-free changes. The Solflex™ XT (extra-thin) discs are made of polyester film and are one-third the thickness of the original paper-based version. This increases stiffness for precise embrasure contouring. The system offers four aluminum oxide grits, ranging from coarse to superfine, and is available in 13 mm (½″) and 9 mm (⅜″) disc diameters (11). In contrast, the OptiDisc is a single-use, four-grit system made of polyethylene, synthetic polymers, aluminum oxide, and epoxy resin. Its discs mount onto a flush-fit, autoclavable mandrel with a patented retention mechanism that ensures a secure attachment that protects adjacent teeth and soft tissues. OptiDisc is indicated for anterior and posterior convex/flat surfaces, interproximal areas, and restorative materials, such as composites, glass ionomers, amalgams, and (semi) precious metals (12). Similarly, the Super-Snap system features four disposable, color-coded discs coated with silicon carbide and aluminum oxide in a metal-free design for contouring, finishing, and polishing direct esthetic resins. The matching Super-Snap Polystrips are double-ended interproximal strips with two grits per strip. The kit also includes Dura-White Stones CA and Composite Fine CA polishers for refining labial, lingual, and occlusal surfaces (13).

Due to resin composite restorative materials' high susceptibility to discoloration, it is essential to evaluate their color stability. Moreover, the accuracy and quality of finishing and polishing techniques, along with control of surface roughness, can influence the color stability and aesthetic outcome of these materials (14, 15). Therefore, the aim of this study is to evaluate the effects of three finishing and polishing kits on the color stability of the Filtek™ Z250 (3M ESPE, St. Paul, MN, USA) resin composite. We hypothesize that varying finishing and polishing protocols will lead to different levels of color changes in composite resins.

## 2 Materials and methods

This laboratory study was conducted at the College of Dentistry, University of Umm Al-Qura, in Makkah, Saudi Arabia. It is also a collaboration with the dental research lab at Imam Abdulrahman Bin Faisal University, College of Dentistry. After ethical approval was obtained from the Institutional Review Board (HAPO-02-K-012-2023-11-1876), the sample size for the study was determined based on a previous study and consisted of 140 specimens (14).

### 2.1 Materials and sample preparation

**Figure 1:**
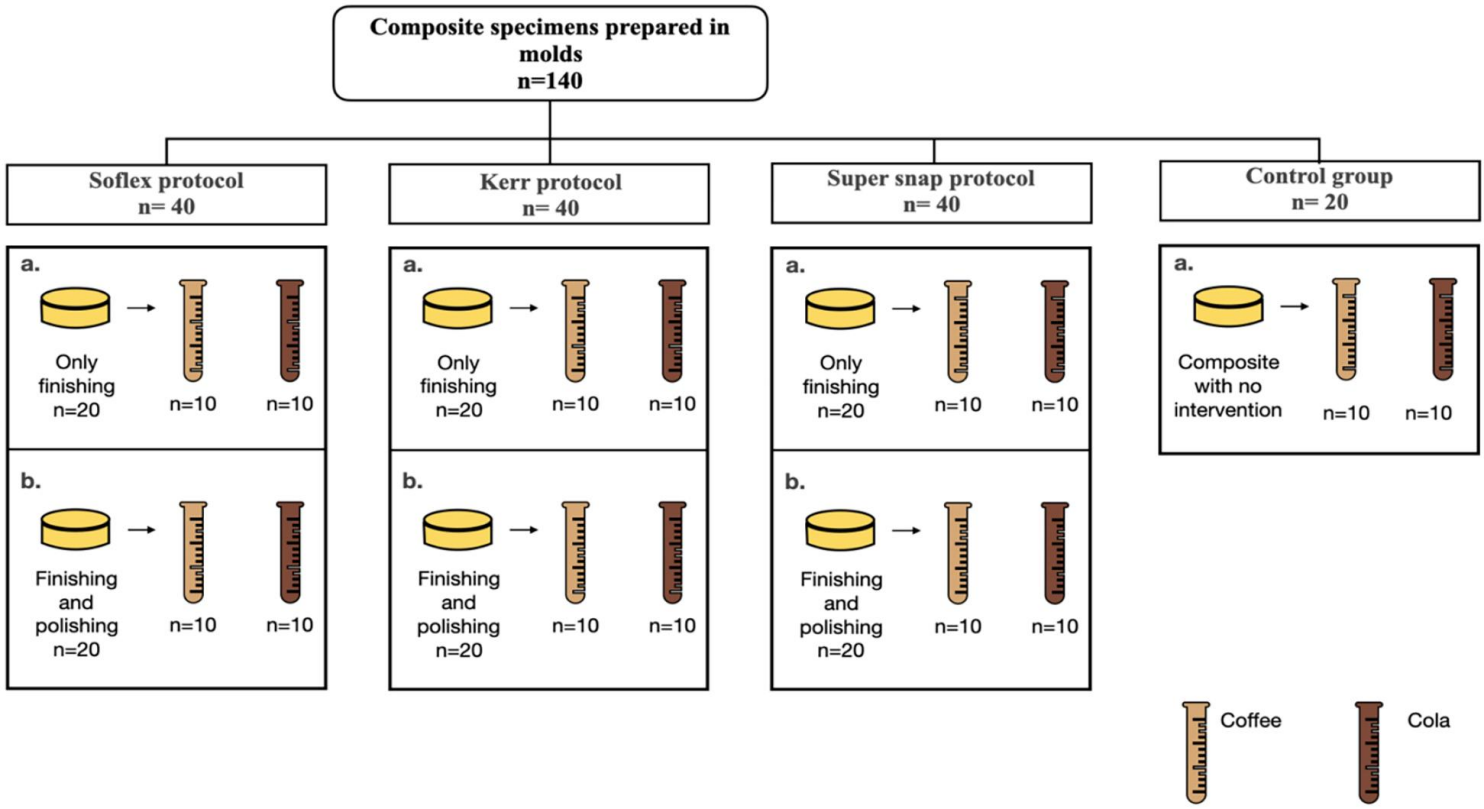
Flow diagram of specimen grouping by finishing and polishing kits headings.

A total of 140 specimens were prepared using a single commercial resin composite Filtek™ Z250 (3M ESPE). Each disc was fabricated using a plastic mold 12 mm in diameter and 2 mm thick (8). The composite was packed into the molds using a plastic instrument, then polymerized by using a dental unit light cure (KAVO) for 40 s for each disc on both top and bottom surfaces against a glass slide. The specimens were divided into four groups: three groups were assigned to three different finishing and polishing kits, with an equal number of specimens in each group (*n* = 40), and one was the control group (*n* = 20). The control group specimens were not subjected to any finishing or polishing procedures (Figure 1).

### 2.2 Finishing and polishing procedures

#### 2.2.1 Sof-Lex™ (3M ESPE, St. Paul, MN, USA)

The kit contains the following discs: coarse, medium 40 μm, fine 24 μm, and super-fine 8 μm. According to the manufacturer's instructions, the super-fine 8 μm disc is used for the final polishing step. The discs were sequentially used in a low-speed handpiece with periodic light pressure and no water cooling. The coarse-grit disc was used for 5 s at a low speed of approximately 10,000 rounds per minute (rpm). After being cleaned with water, the disc was dried by using a 3-in-1 air syringe. This was followed by using the medium-grit disc for 15 s also without water cooling at the same speed. Afterwards, the fine-grit disc was used for 15 s. For the other group, the same steps were followed, in addition to a final polish step using the super-fine grit disc at a low speed for 15 s after further washing and drying.

#### 2.2.2 Super-Snap (Shofu Dental Corporation, Kyoto, Japan)

The kit contains the following grit sizes: black (coarse), violet (medium), green (fine), and red (super-fine). According to the manufacturer, the black (coarse) and violet discs are used for the finishing step, while the green and red discs are used for the polishing step. Discs were sequentially used in a low-speed handpiece with periodic light pressure and no water cooling. The coarse-grit disc (black) was used for 20 s at a low speed of approximately 10,000 rpm. After being cleaned with water, the disc was dried using a 3-in-1 air syringe. This was followed by 20 s of using the medium-grit (violet) disc without water cooling at the same speed. Afterwards, the fine-grit disc (green) was used for 20 s. For the other group after further washing and drying, the same steps were taken in addition to a final polish step using the super-fine grit disc(red) at a low speed for 20 s.

#### 2.2.3 Optidisc (Kerr Dental, Orange, CA, USA)

The kit contains the following discs: coarse, medium, fine, and super-fine, according to the manufacturer. Discs were sequentially used in a low-speed handpiece with periodic light pressure and no water cooling. The coarse-grit disc was used for 20 s at low speed of approximately 10,000 rpm. After being cleaned with water, the disc was dried using a 3-in-1 air syringe. This was followed by 20 s of using the medium-grit disc without water cooling at the same speed. Afterwards, the fine-grit disc was used for 20 s. For the other group, the same steps were taken. After further washing and drying, a final polish step used the super-fine grit disc at a low speed for 20 s.

### 2.3 Solution preparation

The beverages used to immerse the samples in each group were coffee and cola. The coffee solution was prepared by dissolving 2 g of coffee powder (Nescafe Classic, Nestle) in 100 ml of boiled distilled water, according to the manufacturer's recommendation. Each sample was immersed in a separate plastic container containing 5 ml of either prepared coffee solution or cola and stored separately at room temperature at approximate 37°C for a total of 30 days (16). Lids were closed tightly to prevent carbonic gas from escaping and to maintain an appropriate carbonation level. Staining solutions were replaced daily to prevent bacterial colonization and to simulate the daily consumption of these beverages. A previous study suggested that one week of *in vitro* coffee immersion can simulate approximately seven months of *in vivo* exposure. In this study, specimens were immersed in the test solutions for 30 days, representing about 2.5 years of clinical exposure and a simulation of the long-term discoloration effects (17).

### 2.4 Color stability determination

Color measurements of the 140 specimens before and after the immersion in stains were obtained using standard illuminant C and a calibrated spectrophotometer (Color-Eye® 7000 A, X Rite, Carlstadt, NJ, USA) in the visible spectrum range (380–780 nm) (18, 19). The color differences between the specimens were calculated using the following equation:

$$\Delta E_{00}=\sqrt{{\left(\frac{\Delta L^{\prime }}{K_{L}S_{L}}\right)}^{2}+{\left(\frac{\Delta C}{K_{C}S_{C}}\right)}^{2}+{\left(\frac{\Delta H^{\prime }}{K_{H}S_{H}}\right)}^{2}+R_{T}\left(\frac{\Delta C^{\prime }}{K_{C}S_{C}}\right)\left(\frac{\Delta H^{\prime }}{K_{H}S_{H}}\right)}$$

Where *Δ*L′, *Δ*C′, and *Δ*H′ are the differences in lightness, chroma, and hue between the control and modified specimens. RT is a function that accounts for differences in chroma and hue in the blue region, SL, SC, and SH adjust for variations in the location of color differences of samples in L′, a′, and b′ values, while KL, KC, and KH are correction terms set at 2:1:1 (18, 20, 21). *Δ*E00 was assessed on the perceptibility (PT) basis and the acceptability (AT) thresholds were set at 50:50%. PT ranged from 0.8 to 1.30 and 50:50% AT ranged from 1.80 to 2.25 ΔE (22).

### 2.5 Data analysis

Mean color change (ΔE), standard deviation (SD), and 95% confidence intervals were calculated for each group. Normality and homogeneity of variances were verified using the Shapiro–Wilk and Levene's tests, respectively. One-Way ANOVA was conducted to compare mean color changes among the finishing kits, with a significance level set at *α* = 0.05, followed by Tukey's HSD test for *post-hoc* analysis if significant differences were found. All statistical analyses were performed using Stata 16 software.

## 3 Results

**Figure 2:**
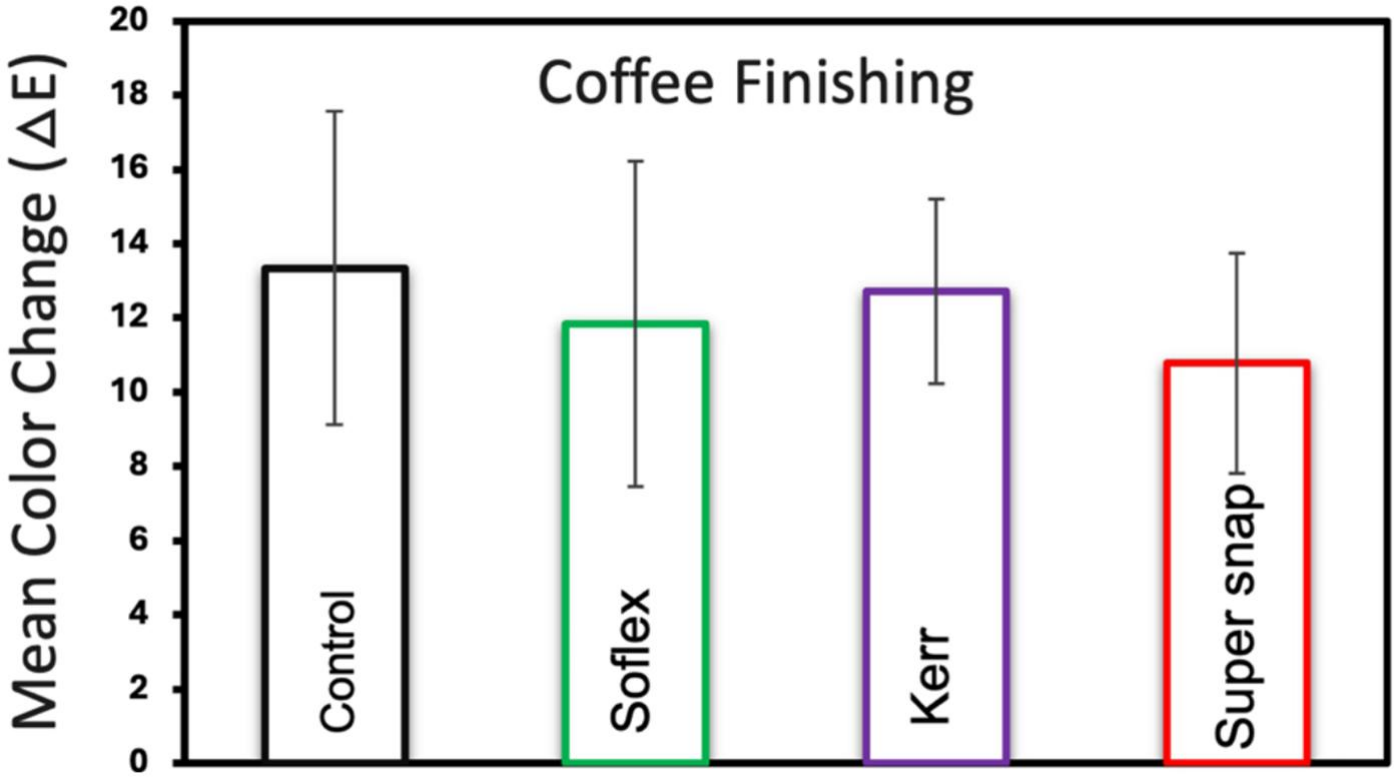
Mean color change and standard deviations of filtek™ Z250 resin composite specimens across various finishing kits in coffee staining medium.

**Figure 3:**
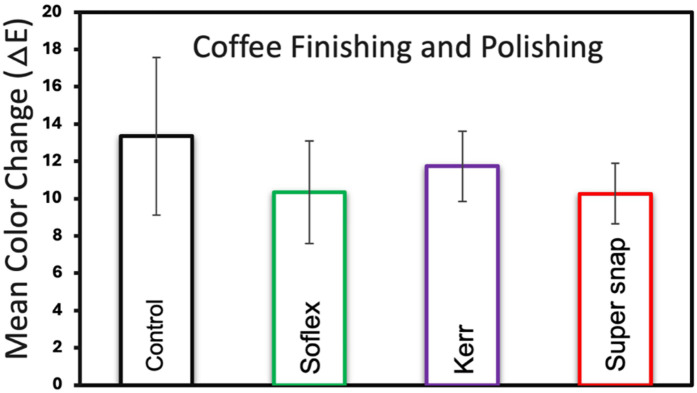
Mean color change and standard deviations of filtek™ Z250 resin composite specimens across various finishing and polishing kits in coffee staining medium.

Figure 2 presents the mean color changes of the Filtek™ Z250 resin composite specimens immersed in coffee staining for different finishing kits. The Super-Snap exhibited the highest color stability with a mean change of 10.7 (±2.96), followed by Sof-Lex at 11.8 (±4.37) and OptiDisc at 12.7 (±2.47). There were no statistically significant differences between the finishing kits (*p* > 0.05). Figure 3 presents the mean color changes of Filtek™ Z250 resin composite specimens immersed in coffee staining for different finishing and polishing kits. After finishing and polishing, the results for color changes improved, with the least color change seen with Super-Snap at 10.2 (±1.62), followed by Sof-Lex at 10.33 (±2.73) and OptiDisc at 11.7 (±1.88). There was no statistically significant difference between the finishing and polishing kits (*p* > 0.05).

**Figure 4:**
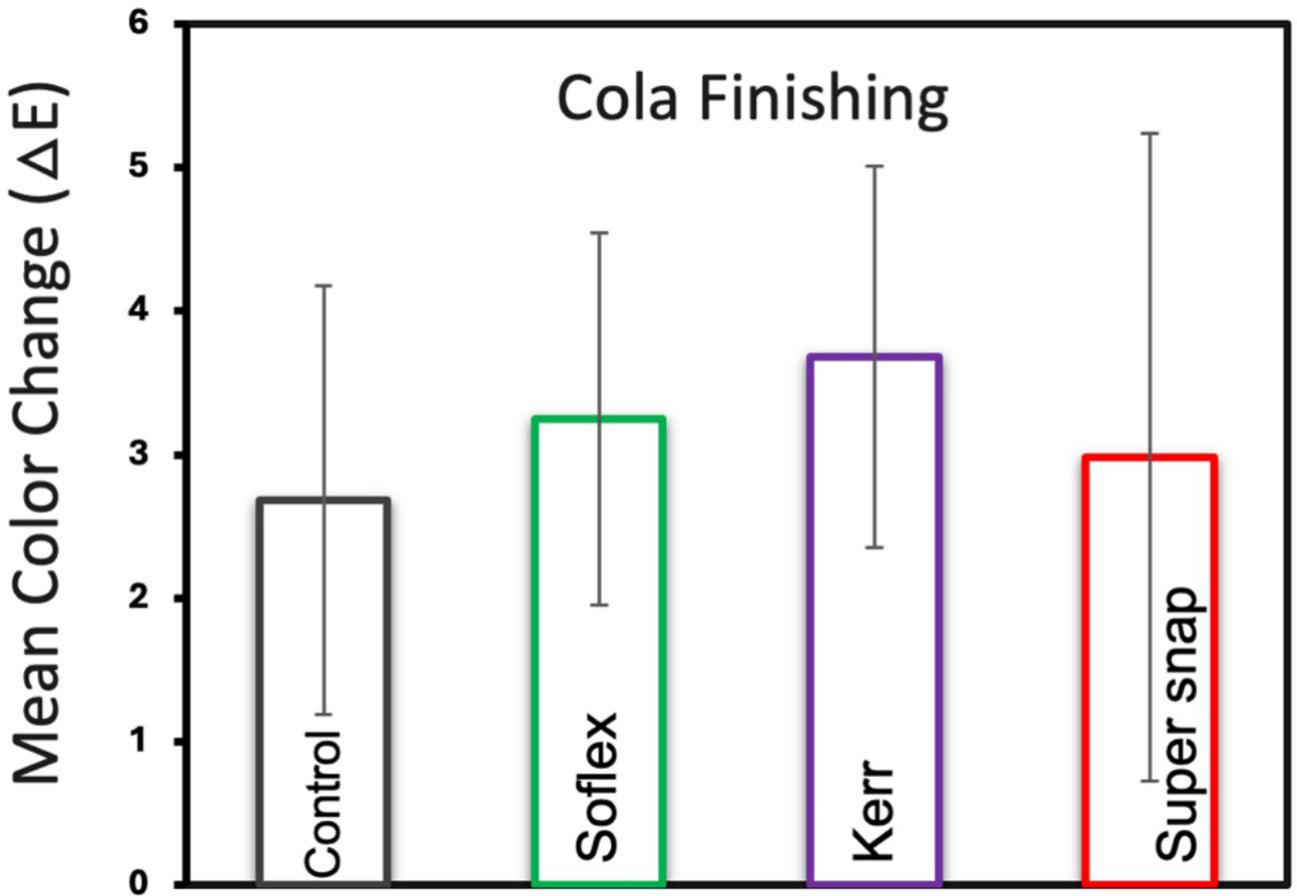
Mean color change and standard deviations of filtek™ Z250 resin composite specimens across Various finishing kits in cola staining medium.

Figure 4 presents the mean color changes of Filtek™ Z250 resin composite specimens immersed in cola staining across various finishing kits. Among the tested kits, the control group exhibited the lowest color changes with a mean of 2.68 (±1.49), followed by Super-Snap at 2.98 (±2.25), Sof-Lex at 3.24 (±1.29), and OptiDisc at 3.68 (±1.33). However, there was no statistically significant difference between the finishing kits (*p* > 0.05).

**Figure 5:**
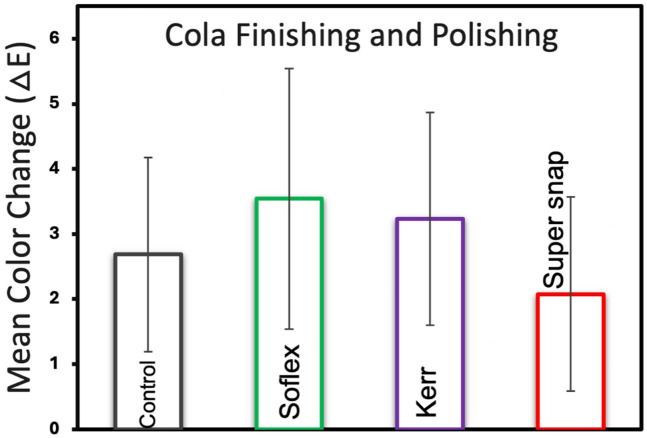
Mean color change and standard deviations of filtek™ Z250 resin composite specimens across Various finishing and polishing kits in cola staining medium.

Figure 5 presents the mean color changes of Filtek™ Z250 resin composite specimens immersed in cola staining across various finishing and polishing kits. After finishing and polishing, the results for color changes improved. The Super-Snap kit exhibited the lowest color change with a mean of 2.07 (±1.49), followed by the control group at 2.68 (±1.49), OptiDisc at 3.23 (±1.63), and Sof-Lex at 3.54 (±2.60). There was no statistically significant difference between the finishing kits (*p* > 0.05).

**Figure 6:**
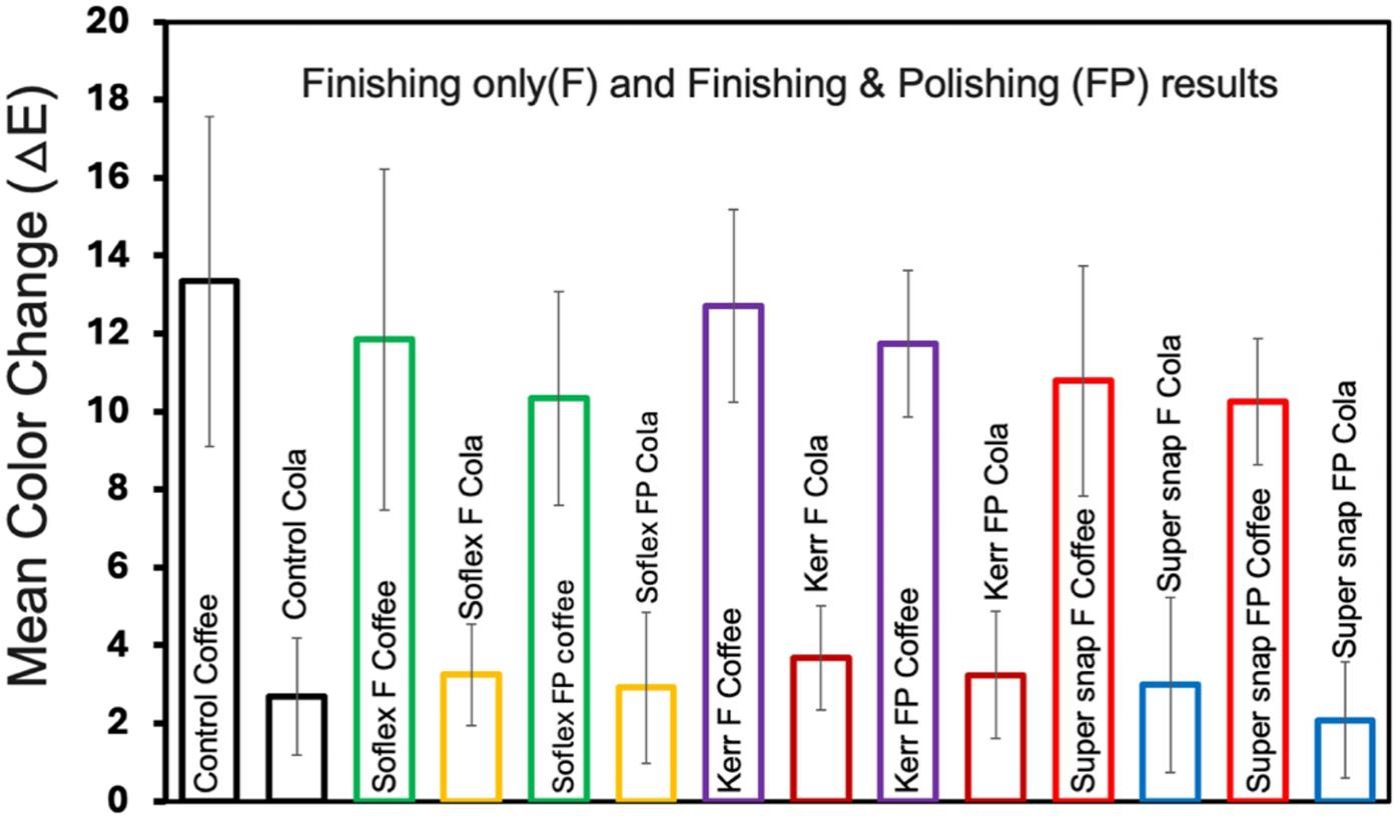
Mean color change and standard deviations of filtek™ Z250 resin. composite specimens across various finishing and polishing kits in coffee and cola staining medium.

Figure 6 compares the color changes of the Filtek™ Z250 resin composite restorations immersed in coffee vs. those immersed in cola and using them both only for finishing and finishing and polishing kits. These results indicate that the coffee media resulted in significantly higher color changes across all groups (*p* < 0.05) compared to cola.

## 4 Discussion

Creating aesthetically pleasing anterior composite restorations has long been challenging due to the variety of staining agents and finishing/polishing techniques that influence color stability (9, 20). The choice of composite polishing and finishing kits is vital in the dental field for enhancing composite restorations' aesthetics and long-term color stability, especially in the anterior area of the mouth (23). This laboratory study examined color changes in Filtek™ Z250 resin composite, using three different finishing and polishing kits, by immersing the specimens in coffee and cola. The results showed that the Super-Snap finishing and polishing kit provided superior color stability for the Filtek™ Z250 resin composite compared to the other kits tested. However, the differences among the three kits were not statistically significant. As a result, the hypothesis of this study was rejected. We had hypothesized that varying finishing and polishing protocols would lead to different levels of color change in composite resins.

These comparable outcomes may be attributed to the similarities in the systems selected for finishing and polishing kits. All systems used in this study were multi-step systems that shared the use of aluminum oxide and silicone-based abrasives in a graduated grit protocol in which the particles progressed from coarse to superfine sizes. This standardized approach created similarly smooth surface finishes by minimizing pigment retention and contributing to the comparable color stability observed. Although the current study kits did not differ significantly, this study reinforces the conclusion that surface smoothness is a key factor. The results clearly illustrate that the finishing and polishing steps are critical and influential. A systematic review revealed that a polishing system's most crucial aspect is the abrasive particle's size. It showed that when the abrasive particle is systematically decreased, the result is the highest polished surface with the lowest surface roughness compared to the single-step systems (24). Another study found that the finishing and polishing methods used can influence the smoothness of a surface, which in turn affects its susceptibility to early discoloration. Rough surfaces tend to retain more stains than smooth ones (25).

In a study assessing bulk-fill composites, 60 discs were tested and showed that finishing and polishing processes influence color stability more than the materials' chemistry (14). Shetty et al. evaluated the impact of polishing techniques and beverage immersion on color stability using samples of nanoceramic composites. Their results showed that polished materials exhibited greater resistance to staining from various beverages compared to the control group (2). Moreover, the color stability of materials is influenced more by the finishing and polishing methods used rather than the material type (24). This phenomenon can be attributed to the fact that stain resistance tends to improve as surface roughness decreases. Therefore, selecting an appropriate finishing and polishing system is crucial for maintaining color stability regardless of the material used (26).

Several studies have examined the performance of various finishing and polishing systems on resin composites, yet the results vary. Alharbi et al. compared the Sof-Lex, Diacomp® Plus Twist, and Enhance®/PoGo® systems using four resin composites, including Filtek™ Z250. They found that Diacomp® produced the smoothest surfaces, followed by Enhance®/PoGo®, while Sof-Lex resulted in the highest surface roughness values. This variation is likely linked to the type and particle size of the abrasives used in each finishing and polishing technique. Both Enhance®/PoGo® and Sof-Lex use aluminum oxide particles during the initial finishing steps. However, the final polishing step differs: Enhance®/PoGo® utilizes a diamond-coated micro-polisher, while Sof-Lex continues with aluminum oxide abrasives. This difference in abrasive composition may explain why Sof-Lex achieved slightly lower surface smoothness than Enhance®/PoGo®. Furthermore, Diacomp® uses diamond-impregnated rubber spiral wheels throughout its polishing sequence, which contributed to the lowest surface roughness values observed. The superior performance of Diacomp® is likely due to the enhanced cutting efficiency and finer finish provided by diamond abrasives compared to aluminum oxide (27). In contrast, Koh et al. evaluated the impact of finishing and polishing systems on the surface roughness of microhybrid and Filtek Supreme nanofill composite resins. They concluded that Sof-Lex discs produced smoother surfaces than Enhance®/PoGo® across all tested materials (28).

Additional studies have assessed the performance of OptiDisc and Super-Snap systems in comparison to other commonly used finishing and polishing kits (29, 30). Dhananjaya et al. found that, although Sof-Lex produced slightly lower surface roughness than Super-Snap, its color stability remained comparable (31). In contrast, Sarıcı et al. reported that OptiDisc consistently achieved lower surface roughness and better color stability than the Enhance®/PoGo® system. This superior performance is likely due to OptiDisc's precise grit progression and uniform distribution of aluminum oxide particles, which enable controlled cutting of both the filler and resin matrix. Overall, standardizing the methodologies used to evaluate the effectiveness of finishing and polishing systems could help resolve these conflicting findings and enhance comparability across studies (32).

Intrinsic and extrinsic factors may cause color instability of composite resin restorations (7). The staining of polymeric materials by colored solutions, including coffee, tea, cola, nicotine, and beverages, has been reported in a study by Ertas et al. It showed that coffee had the highest color change. In contrast, cola showed the lowest color change (25). In line with previous studies, Awliya et al. found no significant differences in color stability among various composite systems following coffee exposure, although coffee induced the highest *Δ*E values across materials. This supports our finding of coffee's pronounced staining effect (33). Similarly, Karaman et al. reported that coffee causes significant discoloration, while cola has a comparably lower impact due to the absence of strong colorants. This aligns with our observation that cola leads to less color changes compared to coffee (34).

In the current study, coffee and cola were used as staining agents because of their frequent intake among the population. Specimens that were immersed in coffee showed the least color stability compared to specimens immersed in cola. Several factors could explain why coffee tends to stain composite resins more than cola. One main factor could be that coffee contains tannins, which are related to a plant-based compound that gives the coffee its dark color and bitter taste. These compounds can create a noticeable color change in the composite resin due to their ability to easily attach to the composite surface (35). Moreover, while both coffee and cola are acidic solutions, coffee is more acidic than cola. This higher acidity makes polymeric materials more prone to staining. It can weaken and soften the resin matrix and cause micro-erosions on the composite surface, allowing the coffee's dark pigment to infiltrate deeply (36).

Finally, coffee is usually consumed hot, which can lead to expansion into the resin's matrix of composites, making them more porous and thus more prone to color changes. These thermal changes can create micro-gaps where pigments from coffee can easily infiltrate and cause discoloration. In contrast, cola is usually consumed cold and does not have the same level of thermal effect, which will reduce its tendency for color changes (37). Another important aspect is the resin composites' structure and the filler particles' characteristics. Past literature has pointed out that the structure of a resin composite and the characteristics of the filler particles have a direct impact on the susceptibility to extrinsic staining (14). Choi et al. reported that the structure and filler particle properties of composites have a direct effect on surface roughness and external colorations (38). However, other studies have concluded that the color stability of resin-based materials is more significantly influenced by the finishing and polishing techniques employed, rather than by the type of filler particles used (24).

The scope of this study is limited by several factors. First, it was conducted in a controlled laboratory environment that does not fully replicate the complexities of the oral cavity, such as fluctuating pH, saliva, and other biological influences. Second, only a single composite material was tested, which limits the generalizability of the results. Future research should compare finishing and polishing protocols with varying numbers of steps and include a broader range of composite materials. It would also be valuable to compare surface roughness outcomes among different finishing and polishing kits. Additional staining media and elements that simulate the oral environment (for example, saliva substitutes, thermal cycling, or biofilm models) should be incorporated to enhance clinical relevance. Despite the finishing and polishing protocols used, the ΔE value exceeded the clinically acceptable threshold (3.3) following coffee exposure. This finding underscores the importance of educating patients with anterior composite restorations about their restorations' susceptibility to staining and the need for regular maintenance and dental follow-ups to preserve aesthetic quality (39).

## 5 Conclusion

Within the limitations of this *in vitro* study, all three multi-step finishing and polishing kits demonstrated comparable color stability in Filtek™ Z250. The greater staining potential of coffee compared to cola underscores the need for clinicians to counsel patients on beverage-related discoloration risks. Regular follow-up visits and professional maintenance are advised to preserve the restorations' integrity and long-term esthetic outcomes.

## Data Availability

The original contributions presented in the study are included in the article/Supplementary Material, further inquiries can be directed to the corresponding author.
